# Disability & Sleep Quality of Custodial Grandparents During Fall 2020 of the COVID-19 Pandemic

**DOI:** 10.1093/geroni/igab046.3687

**Published:** 2021-12-17

**Authors:** Kellie Mayfield, Karen Clark, Raeda Anderson

**Affiliations:** 1 Georgia State University Byrdine Lewis College of Nursing and Health, Atlanta, Georgia, United States; 2 Southern University and A&M College, Baton Rouge, Louisiana, United States; 3 Shepherd Center, Atlanta, Georgia, United States

## Abstract

Disability of custodial grandparents, grandparents who are the primary caretakers of their grandchildren often in parent absent households, are not frequently examined. One in four adults in the U.S. lives with a disability with the highest percentage of disabilities reported in the South. Quality sleep is integral for overall wellbeing and is altered with age. Sleep complaints of older adults are associated with multiple adverse health outcomes such as dementia, stroke and obesity. The objective of this study was to examine the relationship between disability and sleep quality amongst custodial grandparents during the COVID-19, Fall 2019 in Georgia. Thirty-four custodial grandparents were recruited from the Georgia Division of Aging Kinship Care Support Groups, ages 42 to 78, with most identifying as African American. Disability status and the Pittsburgh Sleep Quality Index were measured. Results showed a significant negative relationship for custodial grandparents’ disability status and sleep quality (χ2= 9.167, p=0.027; Γ=-0.683, p=0.002), sleep disturbance (χ2= 12.150, p=0.002; Γ=-0.897, p<.001), and use of sleeping medication (χ2= 9.645, p=0.022; Γ=-0.785, p<.001). Custodial grandparents with a disability had worse sleep quality, more sleep disturbances, and took more sleeping medication compared to custodial grandparents without a disability. Results have implications for kinship care providers and medical practitioners when engaging with custodial grandparents about their health, disability and impacts on their sleep quality.

